# Association of socioeconomic and lifestyle factors with prevalence of diabetes in rural southwest China: a structural equation modelling approach

**DOI:** 10.1136/bmjopen-2024-086050

**Published:** 2024-10-08

**Authors:** Lan Liu, Xia Wu, Guo-Hui Li, Zi-Zi Yu, Du-Li Liu, Allison Rabkin Golden, Xiang-Yang Yin, Le Cai

**Affiliations:** 1 Yunnan Provincial Key Laboratory of Public Health and Biosafety and School of Public Health, Kunming Medical University, Kunming, China; 2 The Second Affiliated Hospital of Kunming Medical University, Kunming, China

**Keywords:** diabetes & endocrinology, China, risk factors, general diabetes

## Abstract

**Abstract:**

**Objectives:**

This study aimed to investigate the prevalence of diabetes using structural equation modelling (SEM) to examine the pathways and associations of socioeconomic and lifestyle factors on diabetes in rural southwest China.

**Design:**

Data were collected from a cross-sectional health interview and examination survey among individuals aged ≥35 years in rural southwest China. Fasting blood glucose, blood pressure, height, weight and waist circumference (WC) were measured for each participant. SEM was employed to assess the relationships between demographic characteristics (sex, age and ethnicity), socioeconomic position (SEP; annual household income, education level and access to medical services), lifestyle factors (obesity status (body mass index and WC) and physical inactivity), hypertension, hyperlipidaemia and family history of diabetes.

**Setting:**

This study was conducted in rural Yunnan Province of China.

**Participants:**

7536 individuals aged ≥35 years consented to participate in the study.

**Results:**

The overall prevalence of diabetes in the present study was 8.3%. Prevalence did not differ by gender (prevalence for both men and women was 8.3% (p>0.05)). The results of SEM indicated that SEP, age, ethnicity, obesity status and physical inactivity had both significant direct and indirect effects on diabetes, with total effect size of 0.091, 0.149, –0.094, 0.212 and 0.089, respectively (p<0.01). Family history of diabetes (0.128, p<0.01), hypertension (0.135, p<0.01) and hyperlipidaemia (0.137, p<0.01) were directly associated with diabetes.

**Conclusions:**

Socioeconomic and lifestyle factors have both direct and indirect effects on prevalence of diabetes in rural southwest China. Future efforts to implement comprehensive interventions to promote the prevention and control of diabetes should in particular focus on obese individuals.

**STRENGTHS AND LIMITATIONS OF THIS STUDY**

- High response rate (96.6%) in this study.
- Our large sample size enhances the validity of our results.
- Haemoglobin A1C was not measured, which could have caused an underestimation of the prevalence of diabetes.

## Introduction

 Diabetes has emerged as a significant public health challenge as its prevalence continues to increase across the globe.^1 2^ According to the International Diabetes Federation, diabetes prevalence was 10.5% (537 million people) in 2021 and is predicted to rise to 12.2% (783 million people) by 2045.^2^ Notably, middle-income countries have witnessed the most substantial relative increases in diabetes prevalence compared with high- and low-income countries.^2^

China, as the world’s largest developing country, has the highest number of diabetics globally: about 141 million people in 2021.^2^ Consistent with global trends, the latest national representative study in China projected diabetes prevalence in adults aged 20–79 years was 8.2% in 2020, but forecast to reach 9.7% by 2030.^3^ These global and China-specific diabetes trends underscore a pressing need for more effective and context-specific risk factor control strategies to prevent or reverse diabetes.

Extensive prior research has estimated the independent contributions of distinct socioeconomic and lifestyle factors to the risk of developing diabetes with conventional regression models.^4 5^ For instance, research has consistently shown that physical inactivity, obesity, high blood lipids and high blood pressure (BP) were correlated with greater odds of diabetes.^4^ Socioeconomic status (SES), commonly assessed by factors such as income and educational attainment,^6^ may have varying effects on diabetes risk across countries with different income levels.^6 7^ In high-income countries, diabetes tends to be more prevalent among populations with lower SES,^6 8^ whereas the opposite is observed in some low- and middle-income countries.^7 9^ Other demographic factors, such as age, gender and ethnicity, also influence diabetes prevalence.^10^

However, solely evaluating the independent direct impact of each socioeconomic and lifestyle variable on diabetes may overlook their potential indirect effects; there are complex inter-relationships between SES, lifestyle factors and diabetes.^11 12^ Structural equation modelling (SEM) is a multivariate technique that allows for simultaneous regression analysis and provides insights into direct and indirect effects of variables.^13^ Despite the potential of SEM, few studies have explored how socioeconomic and lifestyle factors collectively act on multiple pathways leading to diabetes.^14^,^16^ A retrospective cohort study using the Indonesian Family Life Survey data revealed that unhealthy lifestyle behaviours indirectly affected diabetes risk through the mediator of physiological load.^16^ In China, a Wuhan-Zhuhai cohort study also found that low SES had an indirect contribution to the risk of developing diabetes through its mediation of body mass index (BMI), hypertension and triglyceride levels.^17^ However, to date, the literature in China is sparse, and similar studies have not been conducted in rural China.

Yunnan Province is a mountainous region situated on the southwestern frontier of China. It comprises 129 counties, of which most remain underdeveloped. In 2021, Yunnan had a population of 47.2 million, of which approximately half reside in rural areas. In addition, Yunnan maintains the most ethnically diverse population in China, with 25 ethnic groups making up 33.1% of the province’s total population. In Yunnan, diabetes prevalence is high and continues to increase, possibly due to such factors as demographic transition, SES and lifestyle choice.^18^ However, limited research has investigated both direct and indirect effects of socioeconomic and lifestyle factors on diabetes within this resource-limited setting.

Thus, this study aimed to investigate the prevalence of diabetes using SEM to examine the pathways and associations of socioeconomic and lifestyle factors on diabetes in rural southwest China.

## Methods

### Study area and population

We conducted a community-based, cross-sectional health interview and examination survey in three counties of Yunnan Province in 2023. A representative sample of rural residents aged ≥35 years was selected using four-stage stratified random sampling, as described in detail in our previous studies.^18^

### Data collection and measurement

Each participant who gave informed consent was interviewed face to face by a trained interviewer using a pretested and structured questionnaire. Information on demographic characteristics, individual SES (including annual household income, level of education and access to medical services), lifestyle factors (including physical inactivity and obesity status), hypertension, hyperlipidaemia and self-reported family history of diabetes were obtained. Anthropometric measurements (height, weight and waist circumference (WC)), fasting blood glucose (FBG) and BP were also taken.

FBG was measured by physicians from the Second Affiliated Hospital of Kunming Medical University using a FREESTYLE OPTIUM glucometer (Abbott Diabetes Care) by collecting a small drop of peripheral blood in the morning, following an overnight fast of 8–10 hours. Participants who had not fasted at least 8 hours were invited to return for an FBG test the next day after a fast of 8–10 hours.

BP was measured three times consecutively using a mercury sphygmomanometer after participants rested for 5 min in a seated position, following American Heart Association guidelines.^19^ The average of the three BP readings was then recorded.

Weight, height and WC were measured using the standardised procedures recommended by the WHO STEPS manual.^20^ BMI was calculated using an individual’s weight in kilograms divided by his or her squared height in metres.

### Definitions

Diabetes was defined as FBG≥7.0 mmol/L (126 mg/dL), self-reported previous diagnosis of diabetes by a physician or reported use of antidiabetic medications during the 2 weeks preceding the study. Hypertension was defined as average systolic BP ≥140 mm Hg, average diastolic BP ≥90 mm Hg, a self-reported previous diagnosis of hypertension by a physician or reported use of antihypertensive medications in the last 2 weeks preceding the study. Hyperlipidaemia was defined as self-reported previous diagnosis of hyperlipidaemia by a physician, and/or self-reported use of pharmacological lipid-lowering treatment.

BMI was categorised as <18.5, 18.5–24.9, 25.0–27.9 and ≥28.0 kg/m^2^, while WC was classified based on WHO definitions for Asian adults^21^: ≥90 cm in men or ≥80 cm in women. Physical inactivity was defined as engaging in less than 150 min of moderate-intensity activity per week, less than 75 min of vigorous-intensity activity per week or their equivalents, following WHO recommendations for physical activity in adults.^22^

Level of education was categorised into three: illiterate, primary (grades 1–6) and middle (grades 7–9) or higher. Annual household income was also divided into three categories: low (<US$750), medium (US$750–$1200) and high (≥US$1200).

Yunnan ranks among the top three provinces with the poorest spatial accessibility to medical facilities in China.^23^ In rural areas where residents face limited options for health services, travel time to the nearest medical institution is considered as a good measure of accessibility.^24^ Additionally, diabetes prevention programmes within public health services in rural regions of China are implemented by primary healthcare providers, including township hospitals and village clinics. Thus, access to medical services in our study was defined by participants’ walking time from home to the nearest village clinic; a walking time of 30 min or less was defined as good, while >30 min was considered poor.

### Statistical analysis

The research data collected were analysed using descriptive analysis. Categorical variables were expressed as counts and percentages, while continuous variables were presented as mean±SD. The χ^2^ test for independence was used to compare categorical variables, while t-tests were conducted to analyse continuous measures. Confirmatory factor analysis (CFA) was used to assess whether the hypothesised latent variables were well reflected by the involved indicators. SEM was developed to estimate the direct and indirect associations of socioeconomic and lifestyle factors with diabetes. SPSS V.22.0 was used for descriptive analysis and χ^2^ tests, while SEM and CFA were performed using Mplus V.7.4 and fitted by the weighted least squares with the mean and variance adjusted (WLSMV).^16^ In all analyses, a p value <0.05 was considered statistically significant.

**Figure 1:**
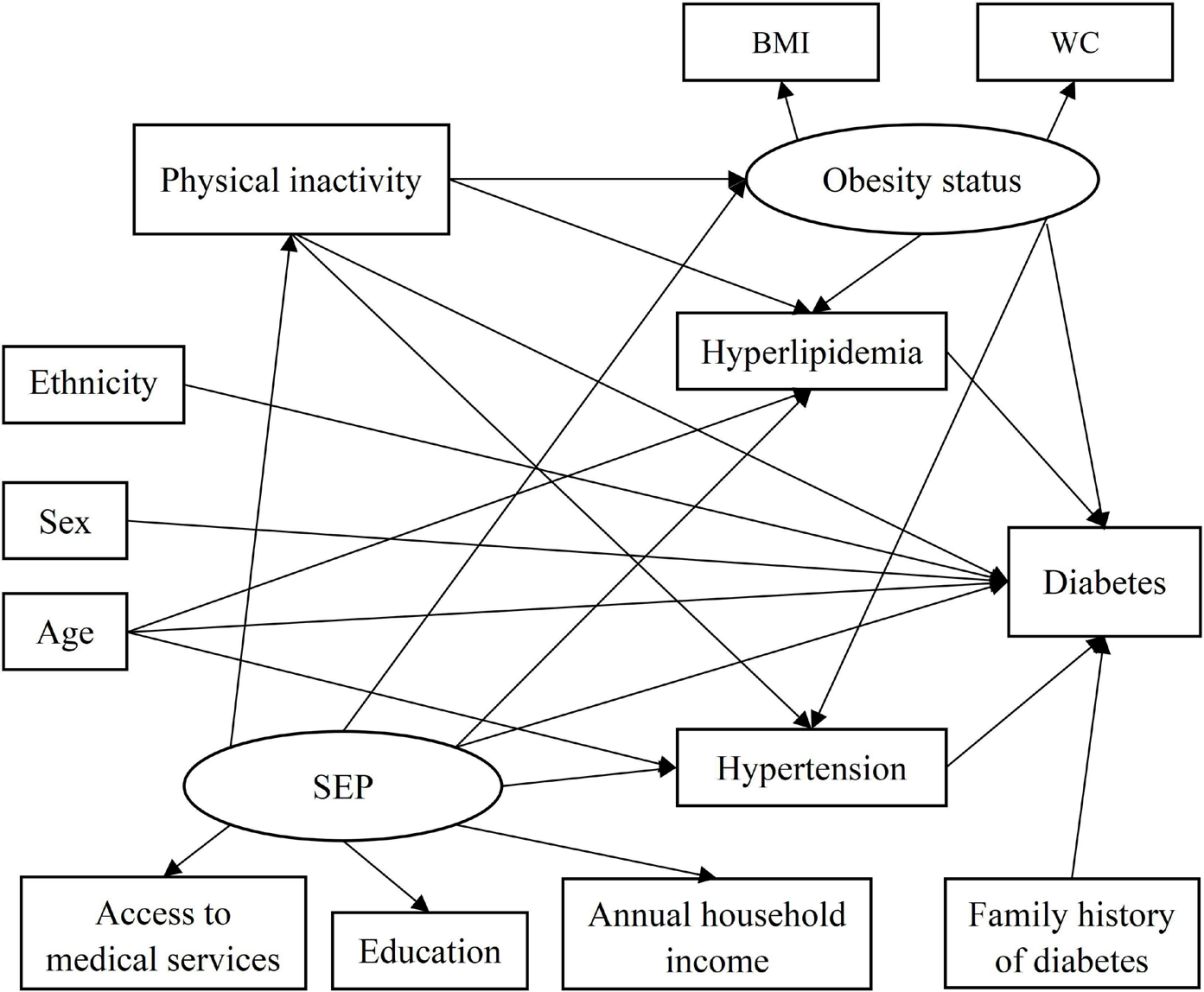
Conceptual model for the association of demographic characteristics (sex, age and ethnicity), SEP (annual household income, education level and access to medical services), lifestyle factors (obesity status (BMI and WC) and physical inactivity), hypertension, hyperlipidaemia and family history of diabetes. BMI, body mass index; SEP, socioeconomic position; WC, waist circumference.

SEM analyses were conducted in two steps. First, a priori conceptual model that specified the paths of demographic, socioeconomic and lifestyle variables, hypertension and hyperlipidaemia on diabetes was developed based on a literature review.^14 16^ The conceptual model developed is pictured in figure 1. In the path diagram, we assumed two latent variables (obesity status (BMI and WC) and socioeconomic position (SEP; annual household income, level of education and access to medical services)) and seven observed variables (sex, age, ethnicity, family history of diabetes, physical inactivity, hypertension and hyperlipidaemia) that directly or indirectly affect diabetes. Latent variables are represented by an ellipse while observed variables are represented by a rectangle. Direct effects are depicted as a single straight arrow directly joining variables, while indirect effects are depicted as arrows passing through one or more mediators. In the second step of SEM analysis, structural equation model parameters were estimated using a WLSMV estimator. The structural model was then modified based on the individual paths’ p values and modification indices (mi) until all paths in the final model were significant and the model fits the observed data well. The criteria for goodness-of-fit indices were root mean square error of approximation (RMSEA) ≤0.08, Comparative Fit Index (CFI) ≥0.90 and Tucker-Lewis Index (TLI) ≥0.90.

### Patient and public involvement

Patients and/or the public were not involved in the design, or conduct, or reporting, or dissemination plans of this research.

## Results

A total of 7800 individuals aged ≥35 years were invited to participate in the present study. Of these, 7536 agreed to participate, representing a response rate of 96.6%. Table 1 summarises the characteristics of the study participants. In total, 3721 males (49.4%) and 3815 females (50.6%) participated. The proportion of ethnic minorities in the study population was 47.0%, and the illiteracy rate was 19.3%. Male participants had higher education levels but lower prevalence of physical inactivity, obesity, central obesity and hyperlipidaemia than female participants (p<0.05). Height, weight, WC and systolic BP were significantly higher in men than in women (p<0.05), while BMI was lower in men than in women (p<0.05).

**Table 1:** Socioeconomic and lifestyle characteristics and mean value of BP, FBG and anthropometric measurements of the study population

Characteristics	Male (n=3721)	Female (n=3815)	All (n=7536)
Age (n, %)			
35–44 years	370 (9.9)	467 (12.2)	837 (11.1)
45–54 years	890 (23.9)	825 (21.6)	1715 (22.8)
55–64 years	982 (26.4)	972 (25.5)	1954 (25.9)
65–74 years	909 (24.4)	929 (24.4)	1838 (24.4)
≥75 years	570 (15.3)	622 (16.3)	1192 (15.8)
Ethnicity (n, %)			
Han	2051 (55.1)	1941 (50.9)	3992 (53.0)
Minority	1670 (44.9)	1874 (49.1)*	3544 (47.0)
Education (n, %)			
Illiterate	380 (10.2)	1071 (28.1)**	1451 (19.3)
Primary (grades 1–6)	1853 (49.8)	1927 (50.5)	3780 (50.1)
Middle (grades 7–9) or higher	1488 (40.0)	817 (21.4)	2305 (30.6)
Annual household income (n, %)			
Low	1145 (30.8)	1218 (31.9)	2363 (31.4)
Medium	757 (20.3)	813 (21.3)	1570 (20.8)
High	1819 (48.9)	1570 (20.8)	3603 (47.8)
Access to medical services (n, %)			
Poor	1291 (34.7)	1346 (35.3)	2637 (35.0)
Good	2430 (65.3)	2469 (64.7)	4899 (65.0)
Physical inactivity (n, %)	1364 (36.7)	1414 (37.1)**	2778 (36.9)
Obesity (n, %)	292 (7.8)	392 (10.3)**	684 (9.1)
Central obesity (n, %)	1505 (40.4)	2146 (56.3)**	3651 (48.4)
Family history of diabetes (n, %)	179 (4.8)	182 (4.8)	361 (4.8)
Hypertension (n, %)	1906 (51.2)	2007 (52.6)	3913 (51.3)
Hyperlipidaemia (n, %)	164 (4.4)	209 (5.5)*	373 (4.9)
Height (cm, mean±SD)	164.8±6.8	153.5±6.5**	159.1±8.5
Weight (kg, mean±SD)	62.6±10.9	54.8±10.3**	58.7±11.3
BMI (kg/m^2^, mean±SD)	23.0±3.4	23.2±3.7*	23.1±3.6
WC (cm, mean±SD)	82.6±9.7	81.2±10.4**	81.9±10.0
Systolic BP (mm Hg, mean±SD)	134.8±21.2	135.4±22.9	135.1±22.1
Diastolic BP (mm Hg, mean±SD)	84.3±12.3	83.7±12.1*	84.0±12.2
FBG (mmol/L, mean±SD)	5.5±1.8	5.6±1.8	5.5±1.8

* P<0.05, **p<0.01.

BMI, body mass index; BP, blood pressure; FBG, fasting blood glucoseWCwaist circumference

Table 2 presents the prevalence of diabetes by socioeconomic and lifestyle factors among the study participants. The overall prevalence of diabetes was 8.3% (8.3% for males and 8.3% for females). Participants of Han ethnicity, higher annual household income and good access to medical services had a higher prevalence of diabetes than their counterparts (p<0.05). Obese, centrally obese, hypertensive, and physically inactive adults, and adults with hyperlipidaemia and family history of diabetes had a significantly higher prevalence of diabetes than their counterparts (p<0.01).

**Table 2:** Prevalence of diabetes by socioeconomic and lifestyle factors in Yunnan Province, China

Characteristics	n	%	P value
Sex			
Male	308	8.3	0.993
Female	316	8.3	
Age (years)			
35–44	34	4.1	<0.01
45–54	110	6.4	
55–64	169	8.6	
65–74	206	11.2	
≥75	105	8.8	
Ethnicity			
Han	402	10.1	<0.01
Minority	222	6.3	
Education			
Illiterate	132	9.1	0.148
Primary (grades 1–6)	290	7.7	
Middle (grades 7–9) or higher	202	8.8	
Annual household income			
Low	158	6.7	0.003
Medium	141	9.0	
High	325	9.0	
Access to medical services			
Poor	191	7.2	0.017
Good	433	8.8	
Physical inactivity			
Yes	308	11.1	<0.01
No	316	6.0	
BMI (kg/m^2^)			
Underweight (<18.5)	22	3.8	
Normal (18.5–24.9)	270	6.4	<0.01
Overweight (25.0–27.9)	233	11.3	
Obesity (≥28.0)	99	14.5	
WC			
Normal	212	5.5	<0.01
Central obesity	412	11.3	
Family history of diabetes			
Yes	120	33.2	<0.01
No	504	7.0	
Hypertension			
Yes	456	11.7	<0.01
No	168	4.6	
Hyperlipidaemia			
Yes	113	30.3	<0.01
No	511	7.1	
All	624	8.3	

BMIbody mass indexWCwaist circumference

**Figure 2:**
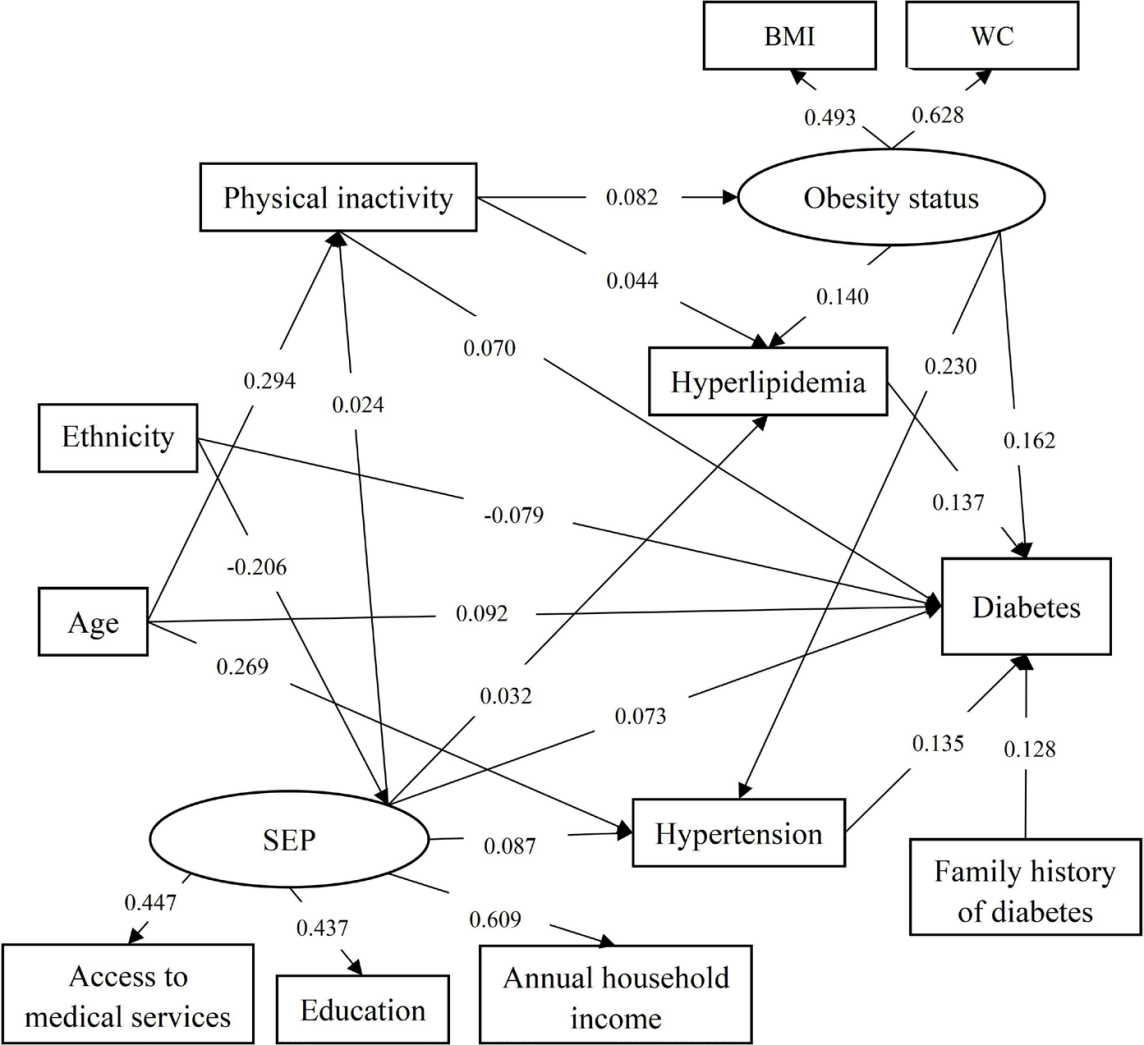
Final structural equation modelling model of associated factors of diabetes among individuals aged ≥35 years in rural southwest China. RMSEA=0.019 (95% CI 0.015 to 0.022), CFI=0.926, TLI=0.901. All path coefficients shown were standardised. BMI, body mass index; CFI, Comparative Fit Index; RMSEA, root mean square error of approximation; SEP, socioeconomic position; TLI, Tucker-Lewis Index; WC, waist circumference.

Figure 2 displays the final SEM model. CFA validated the measurement model for both latent constructs (RMSEA=0.031 (95% CI 0.022 to 0.042), CFI=0.970, TLI=0.925). For the SEP latent variable, the factor loadings were access to medical services (0.447, 95% CI 0.322 to 0.567), education (0.437, 95% CI 0.299 to 0.548) and annual household income (0.609, 95% CI 0.392 to 0.713). For the obesity status latent variable, the factor loadings were BMI (0.493, 95% CI 0.402 to 0.583) and WC (0.628, 95% CI 0.514 to 0.742). All factor loadings are statistically significant (p<0.01). The final SEM model demonstrated an adequate fit to the data, which was determined by the fit index (figure 2; RMSEA=0.019 (95% CI 0.015 to 0.022), CFI=0.926, TLI=0.901). To attain convergence and align effectively with the hypothesis, the following modifications were made for the basic model (figure 1; RMSEA=0.032 (95% CI 0.029 to 0.035), CFI=0.753, TLI=0.657) to construct the optimal final model. First, the statistically insignificant paths were removed from the basic model to determine the most parsimonious final model (p>0.05). Second, based on the mi, the direct effects of age on physical inactivity (mi=368.201) and ethnic on SEP (mi=234.506) were added. Figure 2 illustrates the final SEM model of factors associated with diabetes, with all shown paths being statistically significant (p<0.05). Increasing age had direct effects on physical inactivity (0.294), hypertension (0.269) and diabetes (0.092). Ethnic minority status had a negative association on SEP (−0.206) and diabetes (−0.079). Higher SEP had a positive relationship with hypertension (0.087), hyperlipidaemia (0.032), physical inactivity (0.024) and diabetes (0.073). Physical inactivity showed a positive effect on obesity status (0.082), as did hyperlipidaemia (0.044) and diabetes (0.070). Hypertension (0.137), hyperlipidaemia (0.135) and family history of diabetes (0.128) only had direct effects on diabetes.

Table 3 depicts the direct, indirect and total effects of the studied variables on diabetes. Obesity status had the greatest total effect on diabetes (0.212), followed by age (0.149), hyperlipidaemia (0.137), hypertension (0.135), family history of diabetes (0.128), ethnicity (−0.094), SEP (0.091) and physical inactivity (0.089).

**Table 3:** Direct, indirect and total effects of variables on diabetes

Variable	Path coefficient (95% CI)		
	Direct	Indirect	Total
Age (reference: 35–44 years)	0.092** (0.051, 0.134)	0.057** (0.033, 0.121)	0.149** (0.105, 0.217)
Ethnicity (reference: Han majority)	−0.079** (−0.114, −0.043)	−0.015** (−0.069, −0.017)	−0.094** (−0.131, −0.071)
SEP (reference: low)	0.073** (0.036, 0.109)	0.018** (0.009, 0.035)	0.091** (0.066, 0.150)
Physical inactivity (reference: no)	0.070** (0.042, 0.113)	0.019** (0.013, 0.048)	0.089** (0.054, 0.182)
Obesity status (reference: no)	0.162** (0.110, 0.215)	0.050** (0.014, 0.089)	0.212** (0.137, 0.314)
Family history of diabetes (reference: no)	0.128** (0.085, 0.146)	–	0.128** (0.085, 0.146)
Hyperlipidaemia (reference: no)	0.137** (0.112, 0.163)	–	0.137** (0.097, 0.170)
Hypertension (reference: no)	0.135** (0.097, 0.173)	–	0.135** (0.112, 0.163)

* *p<0.01.

SEPsocioeconomic position

## Discussion

The data in this study indicate a relatively high prevalence of diabetes in rural southwest China. Both individual socioeconomic and lifestyle factors had significant direct and indirect effects on diabetes, in which SEP, age, ethnicity, physical inactivity and obesity status have both direct and indirect effects on diabetes, while family history of diabetes, hyperlipidaemia and hypertension are directly associated with diabetes.

The overall prevalence of diabetes among respondents was 8.3%, which exceeds the average rate of 6.8% in rural China^25^ as well as the rate in rural India (5.2%)^26^ and in Australia (7.7%).^27^ This observed discrepancy may result from a complex interplay of environmental, socioeconomic, lifestyle and other health-related factors.^1 5 7^ Additionally, hypertension, obesity and central obesity were more prevalent in the study region than in other regions of China,^28^ as well as Asian countries such as Bangladesh^29^ and Vietnam.^30^ Hypertension, obesity and central obesity are major risk factors for diabetes.^4^,^6^ The results thus suggest that diabetes remains a major challenge in rural southwest China, and future diabetes prevention and control strategies should incorporate comprehensive lifestyle interventions.

As expected, our results revealed that prevalence of diabetes increases with age,^5 28^ and this association was also mediated by physical inactivity and elevated BP. Based on this finding, we suggest that future initiatives prioritise promoting physical activity and lowering BP in older adults, especially given the growing elderly population in rural China.

Consistent with previous studies, the present study found ethnic disparities in diabetes risk.^10 12^ Specifically, Han Chinese were more likely to develop diabetes than ethnic minority groups. This may be attributed to genetic predisposition and to differences in socioeconomic factors,^6 12^ as the indirect effect of ethnicity on diabetes was mediated by SEP. The findings in this way suggest that interventions targeting those with higher SEP in rural Han majority communities may reduce diabetes risk overall.

This study indicated that high SEP had a positive and direct effect on prevalence of diabetes, which is in line with previous studies in low- and middle-income countries,^7 9 26^ but differs from those in high-income countries.^8^ This may be largely attributable to the economic, demographic and epidemiological shifts occurring in rural China, with higher SEP populations typically the first to adopt unhealthy practices (eg, calorie-dense diets and sedentary lifestyles).^7 31^ Moreover, SEP was positively correlated with hyperlipidaemia and hypertension, thereby strengthening the link between SEP and diabetes. Thus, the findings indicate that individuals with high SEP should be targeted for interventions to reduce diabetes risk.

The present study also indicates that obesity status plays a significant role in the development of diabetes, displaying the highest total effects, a finding in line with previous studies using SEM.^16 17^ Moreover, obesity status indirectly affected diabetes via hypertension and hyperlipidaemia. This may contribute to potential physiopathological mechanisms such as endothelial dysfunction, vascular stiffness and systemic inflammation induced by obesity and central obesity.^32 33^ The findings in this way underscore that effective measures to prevent and manage high BMI and large WC in southwest rural communities could be one of the most effective strategies to prevent diabetes, particularly in adults with hypertension and hyperlipidaemia.

Physical inactivity had significant direct effects on the prevalence of diabetes, as has been established in existing studies.^5 34^ Furthermore, physical inactivity also plays an essential role in increasing diabetes risk factors by exerting a positive effect on hyperlipidaemia, obesity and central obesity. In this context, decreasing sedentariness should be widely promoted in rural southwest China.

Previous research has established that hyperlipidaemia and hypertension have a positive direct impact on developing diabetes.^16 35 36^ Our results are consistent with these studies. This may be due to the fact that elevated levels of blood lipids and elevated BP can lead to insulin resistance, which is the main mechanism of diabetes development.^5 36^ Moreover, in the final SEM in our study, hyperlipidaemia and hypertension were also found to be the most common mediators connecting socioeconomic and lifestyle factors to diabetes status. Hence, from a public health perspective, active prevention and control of hypertension and hyperlipidaemia can be pivotal for diabetes prevention.

Family history of diabetes was also a risk factor for diabetes that had a direct impact on its prevalence. While genetic factors are non-modifiable, it is possible to reduce diabetes by regularly monitoring blood glucose and executing health education campaigns to advocate for healthy behaviours, especially for those with a family history of diabetes.^37^

Based on our results, the following strategies may have the potential to improve control and prevention of diabetes in rural China: (1) prioritise addressing obesity status (including large BMI and WC), high BP and high blood lipids as primary public health efforts for diabetes prevention; (2) promote increased physical activity as a fundamental preventive measure against diabetes among all demographic groups; and (3) implement targeted educational campaigns on healthy lifestyle choices and regular health screenings, specifically tailored to individuals with high SEP.

The following limitations of this study should be noted. First, we performed a cross-sectional study, so the causality of the reported associations cannot be determined. Second, diagnoses of diabetes were solely based on FBG test, and this may underestimate the actual prevalence of diabetes. Also, our estimates were not distinguished by diabetes type; however, given that the current research focuses on adults, the prevalence rates primarily reflect type 2 diabetes. Third, data on diet, sleep, smoking and drinking were not included in the study, and these could be important factors in influencing diabetes prevalence. Finally, the present findings were based on a study of a random sampling of three counties, which may limit the ability to generalise the results to the whole province.

In conclusion, the findings of this study suggest that rural southwest China should place a greater emphasis on preventing and managing diabetes, and future interventions on diabetes should take into account individual socioeconomic factors in parallel with lifestyle factors.

## Data Availability

Data are available upon reasonable request.
