# Supplementary material for: Association of socioeconomic and lifestyle factors with prevalence of diabetes in rural southwest China: a structural equation modelling approach
Source: BMJ Open. 2024 Oct 8;14(10):e086050. doi: 10.1136/bmjopen-2024-086050 (PMC11474682; doi:10.1136/bmjopen-2024-086050)
Supplement: Supplementary file 1 [file bmjopen-2024-086050.reviewer_comments.pdf]

PEER REVIEW HISTORY

BMJ Open publishes all reviews undertaken for accepted manuscripts. Reviewers are asked to complete a checklist review form and are provided with free text boxes to elaborate on their assessment. These free text comments are reproduced below.

ARTICLE DETAILS

Title (Provisional)

Association of socioeconomic and lifestyle factors with prevalence of diabetes in rural southwest China：a structural equation modeling approach

Authors

LIU, Lan; WU, Xia; LI, Guo-hui; YU, Zi-zi; LIU, Du-li; RABKIN GOLDEN, Allison; YIN, Xiang-yang; Cai, Le

---

VERSION 1 - REVIEW

**Reviewer**	**1**
**Name**	**Duan, Ming-Jie**
**Affiliation**	**Wageningen University & Research, Human Nutrition & Health**
**Date**	**18-Apr-2024**
**COI**	**There are no competing interests.**

Thanks for inviting me to review this manuscript. The authors investigated the interrelationships between multiple risk factors on prevalence diabetes in rural China in Yunnan province. The manuscript is in general well-written. However, I have the following comments:

(1) Throughout the manuscript, no distinguishment between type 1 and type 2 diabetes was made. I understand this may be due to the limitation of the survey data. I was wondering, do the authors have some knowledge about the prevalence of type 1 diabetes in rural China? Will type 1 diabetes influence the study results?

(2) Some terminologies used in the manuscript should be consistent with each other. For example, I see that “height, weight, and waist circumference” was used in the Abstract Methods, while “body fat distribution” and “central obesity” were used in Results and Conclusions in the Abstract. Please make clear which indicators are used to measure body fat distribution / central obesity.

(3) If in the Abstract Methods the authors chose to describe which indicators were measured, please make them complete. For example, hyperlipidemia was mentioned in the Results of the Abstract, but this info is missing in the Methods in the Abstract.

(4) On Page 7, please clarify why consider hypertension, obesity, and hyperlipidemia as lifestyle factors. These factors are generally considered as clinical biomarkers and are hypothesized (also in this paper) to be directly influenced by physical inactivity (lifestyle factor).

(5) In the analysis, the authors categorized some variables into dichotomous variables, such as income into high vs low, and the waist circumference by cut-offs. This practice may lose information and variation of the data. In addition, I was surprised to see that education level was only categorized into illiterate and having any education. However, it is clearly known that having different levels of education may also impact on lifestyle factors, clinical biomarkers, and health outcomes. Please elaborate the choice for this.

(6) Please elaborate why constructed a latent variable for SEP, rather than analyzing their effects separately. It has been shown in previous studies that education, income, and access to medical service may affect health through different mechanisms.

(7) Access to medical services was defined by participants' walking time from home to the nearest village clinic. I would encourage the authors to provide more background information on this. How is this primary medical service system operated/organized in China. Are there prevention programs implemented by them? Do people prefer to go to higher-level "SanJia" hospital in the area? In addition, why do the authors consider this factor as a socio-economic status factor.

(7) The authors used AMOS to conduct the SEM analysis. Please specify which estimation method was used to deal with ordinal data? The commonly used maximum likelihood approach may lead to biased estimates if there are ordinal variables in the model.

(8) Are modification indices considered to improve the model?

(9) The authors are encouraged to further elaborate on their reasons for removing several paths. For example, on page 11, the effects of age on hyperlipidemia and diabetes were removed because the path estimates were not significant. This seems an odd choice, as it violates the fundamental "biological principles" and also the well-established evidence, but only with the aim to achieve a "significant" model. Please justify this choice. In addition, I would also encourage the authors to provide more details on how the final models were achieved.

(10) A fine-tuned discussion on policy implications are needed. The authors have mentioned several potential targets, but these targets are from different ecological levels, which also requires different strategies to tackle them. As shown in the results, socio-economic and demographic factors play an important role in the hypothesized paths to developing

diabetes. If putting these results into the local context, how should the local governments and health authorities translate the findings from this paper to real-life practice?

(11) Information on ethics approval is missing.

**Reviewer**	**2**
**Name**	**Wu, Wenwen**
**Affiliation**	**Hubei University of Medicine, School of health science**
**Date**	**28-Apr-2024**
**COI**	**No.**

1.Since lifestyle is included, why are some factors not included, such as smoking, drinking, etc. Please state the reason. It is necessary to supplement the research limitations.

2.Please add the ethics review number.

3.The overall prevalence of diabetes among respondents was 8.3%, which exceeds the average rate of 6.8% in rural China as well as the rate in rural India (5·2%) 24 and in Australia (7.7%) . Please discuss the reasons.

VERSION 1 - AUTHOR RESPONSE

Reviewers' comments:

Reviewer: 1

Dr. Ming-Jie Duan, Wageningen University & Research

Comments to the Author:

Thanks for inviting me to review this manuscript. The authors investigated the interrelationships between multiple risk factors on prevalence diabetes in rural China in Yunnan province. The manuscript is in general well-written. However, I have the following comments:

(1) Throughout the manuscript, no distinguishment between type 1 and type 2 diabetes was made. I understand this may be due to the limitation of the survey data. I was wondering, do the authors have some knowledge about the prevalence of type 1 diabetes in rural China? Will type 1 diabetes influence the study results?

Response: Thank you for addressing this point. Indeed, due to limitations in the survey data, we did not distinguish between type 1 and type 2 diabetes throughout the manuscript. However, data on the prevalence of type 1 diabetes in rural China are limited, and existing few studies suggest it constitutes a smaller proportion compared to type 2 diabetes in this setting. Moreover, the incidence of type 1 diabetes peaks in childhood, while the incidence of type 2 diabetes in adults is an order of magnitude higher and peaks in middle and older ages. As our study focuses on adults (aged ≥35 years), the prevalence rates mainly indicate type 2 diabetes. Also, we've noted this limitation in the Discussion section, Page 13.

(2) Some terminologies used in the manuscript should be consistent with each other. For example, I see that "height, weight, and waist circumference" was used in the Abstract Methods, while "body fat distribution" and "central obesity" were used in Results and Conclusions in the Abstract. Please make clear which indicators are used to measure body fat distribution / central obesity.

Response: Done as what you have suggested. Please see Abstract, Method section.

(3) If in the Abstract Methods the authors chose to describe which indicators were measured, please make them complete. For example, hyperlipidemia was mentioned in the Results of the Abstract, but this info is missing in the Methods in the Abstract.

Response: Done as what you have suggested. Please see Abstract, Method section.

(4) On Page 7, please clarify why consider hypertension, obesity, and hyperlipidemia as lifestyle factors. These factors are generally considered as clinical biomarkers and are hypothesized (also in this paper) to be directly influenced by physical inactivity (lifestyle factor).

Response: Thanks for your good suggestions! We have removed hypertension and hyperlipidemia from the lifestyle factors. While hypertension, obesity, and hyperlipidemia are typically regarded as clinical biomarkers, actively losing weight is recognized as components of healthy lifestyle behaviors. This aligns with the broader concept of lifestyle health factors, therefore obesity status is still classified as one of lifestyle factors in our study. Please see Page 7, Methods, Data collection and measurement section.

(5) In the analysis, the authors categorized some variables into dichotomous variables, such as income into high vs low, and the waist circumference by cut-offs. This practice may lose information and variation of the data. In addition, I was surprised to see that education level was only categorized into illiterate and having any education. However, it is clearly known

that having different levels of education may also impact on lifestyle factors, clinical biomarkers, and health outcomes. Please elaborate the choice for this.

Response: Thanks for your good comments! We have re-categorized these variables to avoid loose information. Please see Page 7, Method, Definitions section.

(6) Please elaborate why constructed a latent variable for SEP, rather than analyzing their effects separately. It has been shown in previous studies that education, income, and access to medical service may affect health through different mechanisms.

Response: Numerous studies have explored how socioeconomic indicators relate to diabetes in rural China, but few research has addressed the impact of an individual's SEP on developing diabetes. Our study aimed to fill this gap by constructing a latent variable for SEP, enabling a comprehensive analysis of its impact on diabetes.

(7) Access to medical services was defined by participants' walking time from home to the nearest village clinic. I would encourage the authors to provide more background information on this. How is this primary medical service system operated/organized in China. Are there prevention programs implemented by them? Do people prefer to go to higher-level "SanJia" hospital in the area? In addition, why do the authors consider this factor as a socio-economic status factor.

Response: China launched the National Essential Public Health Service Package (NEPHSP) since 2009, which defines a minimum set of public health services to be available for all citizens. These public health services are delivered by primary healthcare (PHC) providers, which include township hospitals and village clinics in rural regions. Within this framework, diabetes prevention programs are implemented, encompassing screening, monitoring, routine follow-up, and tailored interventions for individuals aged 35 years and older.

Tertiary hospitals, such as "SanJia" hospitals, offer comprehensive services and specialized treatments suitable for complex medical conditions. However, in economically underdeveloped regions of central and western China, particularly vast rural areas, township hospitals and village clinics are preferred due to shorter travel times and lower out-of-pocket costs compared to higher-level hospitals.

In Yunnan, among China's provinces with the poorest spatial accessibility to medical facilities, travel time to the nearest medical institution is crucial, particularly in rural areas with limited healthcare options. Socio-economic status can influence where individuals live, which in turn affects their proximity to medical facilities. Higher SES individuals typically have better access to healthcare resources compared to those with lower SES, where healthcare

facilities may be scarce. Therefore, considering access to medical services as a socio-economic status factor is appropriate. We have added some sentences to explain it. Please see Page 7, Method, Definitions section.

(8) The authors used AMOS to conduct the SEM analysis. Please specify which estimation method was used to deal with ordinal data? The commonly used maximum likelihood approach may lead to biased estimates if there are ordinal variables in the model.

Response: Thank you for your thoughtful review and insightful comments! We carefully considered the potential bias associated with utilizing the maximum likelihood approach with ordinal variables. And we have switched to the WLSMV method to handle the ordinal data in the SEM model using Mplus version 7.4, as it provides relatively robust estimates for ordinal variables. Please see Page 8, Method, Statistical analysis section.

(9) Are modification indices considered to improve the model?

Response: Yes, modification indices are utilized to propose potential adjustments to the initial model, aiming to improve its fit to the data. Please see Page 10, Result section, paragraph5.

(10) The authors are encouraged to further elaborate on their reasons for removing several paths. For example, on page 11, the effects of age on hyperlipidemia and diabetes were removed because the path estimates were not significant. This seems an odd choice, as it violates the fundamental “biological principles” and also the well-established evidence, but only with the aim to achieve a “significant” model. Please justify this choice. In addition, I would also encourage the authors to provide more details on how the final models were achieved.

Response: We have refined the model estimation methodology and adapted the analysis software used in our study. And we have re-provided additional details on how the final models were achieved. Please see Page 10, Result section, paragraph 5.

(11) A fine-tuned discussion on policy implications are needed. The authors have mentioned several potential targets, but these targets are from different ecological levels, which also requires different strategies to tackle them. As shown in the results, socio-economic and demographic factors play an important role in the hypothesized paths to developing diabetes. If putting these results into the local context, how should the local governments and health authorities translate the findings from this paper to real-life practice?

Response: Thanks for your precious suggestions. Based on our findings, several strategies have been proposed that may help local governments and health departments better manage and control diabetes. Please see Page 13, Discussion section, paragraph 10.

(12) Information on ethics approval is missing.

Response: Apologies for the oversight. We have included the necessary details on ethics approval. Please see Page 14, Ethical approval and consent to participate section.

Reviewer: 2

Prof. Wenwen Wu, Hubei University of Medicine

Comments to the Author:

1.Since lifestyle is included, why are some factors not included, such as smoking, drinking, etc. Please state the reason. It is necessary to supplement the research limitations.

Response: Thanks for your valuable comment! Unfortunately, we don't have these data in our study. We have added it as one of limitations in our study. Please see Page 13.

2.Please add the ethics review number.

Response: Done as what you have suggested. Please see Page 14, Ethical approval and consent to participate section.

3.The overall prevalence of diabetes among respondents was 8.3%, which exceeds the average rate of 6.8% in rural China as well as the rate in rural India (5·2%) 24 and in Australia (7.7%). Please discuss the reasons.

Response: Done as what you have suggested. Please see Page 11, Discussion section, paragraph 2.

VERSION 2 - REVIEW

**Reviewer**	**1**
**Name**	**Duan, Ming-Jie**
**Affiliation**	**Wageningen University & Research, Human Nutrition & Health**
**Date**	**12-Jun-2024**

COI **I have no competing interests.**

Thank you for inviting me to review this revised manuscript. The authors have thoroughly addressed the comments, and the quality of the manuscript has been substantially improved. However, there remain some minor points that need further clarification.

1. In the Abstract Results, the authors wrote that "ethnicity had an effect on diabetes, with total effects of …". Please specify which ethnic group is the reference group. There are also several other cases like this in the manuscript (such as in Table 3).

2. In the Abstract Conclusions, the authors listed every factor analyzed in the model as potential prevention targets, while this provides no added value to what is known in the field, as these are commonly known risk factors for type 2 diabetes. One of the advantages of using SEM (especially in this case) is to quantify the interrelationships among these risk factors in the population of interest. By comparing their effect sizes, priority prevention targets (with the largest effect on type 2 diabetes risk) can be identified in this population. I recommend the authors provide tailored recommendations based on the results specifically for this population.

3. The statement "The findings could provide practical guidance …" is not a strength.

4. The authors applied principal component analysis (PCA) to construct the latent variable for socioeconomic position (SEP). I was wondering, is this method also used for the latent variable of obesity status? And why did the authors choose two different methods in constructing latent variables in this model? In Figure 1, SEP and obesity status are illustrated in a reflective manner (the single-headed arrows flow from a reflective latent SES construct to the indicators), and I would also expect that these two latent variables were analyzed as such. I am ignorant about how a PCA latent variable should be displayed in the conceptual model in this case, but the statistics of PCA surely differ from the statistics of the reflective construct of a latent variable. I recommend the authors check this. In addition, I missed the factor loadings of the two obesity indicators on the latent variable in the manuscript.

5. Please provide confidence intervals or standard errors of the estimates, in addition to the significance indicated by P-values.

6. There is a small typo on Page 11, line 33: 0.0.32.

7. Did the physical activity level assessed include occupational physical activity?

8. The authors concluded that diabetes prevention should target the high SEP population. However, Table 2 clearly shows that the prevalence of diabetes was higher in both illiterate (9.1%) and middle or higher education (8.8%) groups. This contradicts the general conclusions made. Please further clarify this.

9. Please provide details of the PCA used to construct SEP in the manuscript (can also be in supplementary files), including the factor loadings of each SEP indicator. This is highly important for the transparency of the analysis.

VERSION 2 - AUTHOR RESPONSE

Reviewers' comments:

Reviewer: 1

Dr. Ming-Jie Duan, Wageningen University & Research

Comments to the Author:

Thank you for inviting me to review this revised manuscript. The authors have thoroughly addressed the comments, and the quality of the manuscript has been substantially improved. However, there remain some minor points that need further clarification.

1. In the Abstract Results, the authors wrote that "ethnicity had an effect on diabetes, with total effects of …". Please specify which ethnic group is the reference group. There are also several other cases like this in the manuscript (such as in Table 3).

Response: Done as what you have suggested. Please see Page 9, Result section, paragraph 3 and Page 21, Table 3.

2. In the Abstract Conclusions, the authors listed every factor analyzed in the model as potential prevention targets, while this provides no added value to what is known in the field, as these are commonly known risk factors for type 2 diabetes. One of the advantages of using SEM (especially in this case) is to quantify the interrelationships among these risk factors in the population of interest. By comparing their effect sizes, priority prevention targets (with the largest effect on type 2 diabetes risk) can be identified in this population. I recommend the authors provide tailored recommendations based on the results specifically for this population.

Response: Thank you for your valuable suggestions. We've revised the conclusions in the Abstract to offer tailored recommendations based on our SEM analysis. Please see Abstract, Conclusions section.

3. The statement "The findings could provide practical guidance …" is not a strength.

Response: Done as what you have suggested. Please see Page 3, Strengths and limitations of this study section.

4. The authors applied principal component analysis (PCA) to construct the latent variable for socioeconomic position (SEP). I was wondering, is this method also used for the latent

variable of obesity status? And why did the authors choose two different methods in constructing latent variables in this model? In Figure 1, SEP and obesity status are illustrated in a reflective manner (the single-headed arrows flow from a reflective latent SES construct to the indicators), and I would also expect that these two latent variables were analyzed as such. I am ignorant about how a PCA latent variable should be displayed in the conceptual model in this case, but the statistics of PCA surely differ from the statistics of the reflective construct of a latent variable. I recommend the authors check this. In addition, I missed the factor loadings of the two obesity indicators on the latent variable in the manuscript.

Response: Thank you for your detailed review and constructive comments! We did not use PCA to construct the latent variable for obesity status. Instead, we employed SEM, specifically confirmatory factor analysis (CFA), to model the latent variable for obesity status. This approach has been updated in the manuscript to reflect this change. Please see Page 8, Method, Statistical Analysis section.

We acknowledge that PCA and SEM involve different statistical properties and interpretations for latent variables. To address this, we have switched from PCA to SEM to construct the SEP latent variable. This ensures methodological consistency and clarity in analyzing interrelationships with other variables. We have also carefully analyzed the factor loadings of each indicator and ensured that they reflect a meaningful and interpretable underlying construct of these two latent variables. Please see Page 9, Result section, paragraph 3.

Finally, we have included the factor loadings for the obesity indicators on the latent variable in the revised manuscript. Please see Figure 2 for the updated results.

5. Please provide confidence intervals or standard errors of the estimates, in addition to the significance indicated by P-values.

Response: Done as what you have suggested. Please see Page 21, Table 3.

6. There is a small typo on Page 11, line 33: 0.0.32.

Response: Sorry for the mistake. We have replaced “0.0.32” by “0.032”. Please see Page 11, line 33.

7. Did the physical activity level assessed include occupational physical activity?

Response: Thank you for your question. Yes, our study did include occupational physical activity in the assessment. Given the fact that our participants were primarily engaged in farming, their daily physical activity levels naturally encompassed the physical demands of their work, including fieldwork, heavy lifting, and prolonged standing or walking. As such,

the physical activity levels assessed in our study reflected not only leisure-time activities but also the occupational demands inherent to their profession.

8. The authors concluded that diabetes prevention should target the high SEP population. However, Table 2 clearly shows that the prevalence of diabetes was higher in both illiterate (9.1%) and middle or higher education (8.8%) groups. This contradicts the general conclusions made. Please further clarify this.

Response: Thank you for your insightful comment. We appreciate the opportunity to clarify this discrepancy. Table 2 indeed shows that the prevalence of diabetes is slightly higher among individuals with illiteracy (9.1%) and those with middle or higher education (8.8%) compared to those with primary education. However, statistical analysis did not reveal significant differences in diabetes prevalence across these education levels, indicating that we cannot confidently conclude that the prevalence of diabetes differs significantly across education levels. Our recommendation to focus diabetes prevention efforts on high SEP populations is informed by the prevalence data alongside SEM analyses. Specifically, the SEM analyses demonstrated a positive association between high SEP and diabetes risk, supporting our conclusion that high SEP populations are at elevated risk. We hope this clarification addresses the discrepancy and provides a clearer rationale for our conclusions.

9. Please provide details of the PCA used to construct SEP in the manuscript (can also be in supplementary files), including the factor loadings of each SEP indicator. This is highly important for the transparency of the analysis.

Response: Thank you for your good comments! We would like to clarify that after careful consideration and further analysis, we have decided to rely on SEM to construct the SEP latent variable. The factor loadings for each SEP indicator were detailed in the revised manuscript. Please see Page 23, Figure 2.

VERSION 3 - REVIEW

**Reviewer**	**1**
**Name**	**Duan, Ming-Jie**
**Affiliation**	**Wageningen University & Research, Human Nutrition & Health**
**Date**	**25-Aug-2024**
**COI**	**I have no competing interests.**

I have no further comments.
